# Optical Assets of *In situ* Electro-assembled Platinum Black Nanolayers

**DOI:** 10.1038/s41598-017-14630-3

**Published:** 2017-11-02

**Authors:** S. E. Stanca, F. Hänschke, G. Zieger, J. Dellith, A. Ihring, A. Undisz, H.-G. Meyer

**Affiliations:** 1 Leibniz Institute of Photonic Technology, Albert-Einstein-Straße, 9, 07745 Jena, Germany; 2 Otto Schott Institute of Materials Research, Friedrich Schiller University, Löbdergraben 32, 07743 Jena, Germany

## Abstract

Optoelectronic technology has been increasingly driven towards miniaturization. In this regard, maintaining the optical properties of the bulk materials while reducing their size is a critical need. How thin must the film be to preserve the bulk material´s optical absorbance and reflectance characteristics? This is the central question for our study of the *in situ* electro-assembly broad band optical absorber films of platinum in non-aqueous solution of PtCl_4_. By reducing the *in situ* constructed film to sub-visible-wavelength thicknesses, the measured reflectance in the region from the ultraviolet to the infrared remained close to that exhibited by the micrometre-width films. These platinum black films broadly absorb electromagnetic waves at a sub-incident-wavelength thickness owing to their plasmonically increased absorbance cross-section. Simulation of various incident energy electron trajectories gives insights into the electron depth through the porous platinum black of ρ = 1.6 g/cm^3^ and previews the optical behaviour close to the atomic thickness.

## Introduction

Plasmonic nanostructures have unlocked a new gate in the construction of efficient optical absorber nanoscale films with similar absorbance characteristics to the classical micrometre-size thick triple layer absorber^1–3^. Based on plasmon theory, the expanded absorbance cross-section of the miniaturized structure creates the expectation of beneficial optical properties. Upon interaction with light, the conduction electrons of noble metal structures with dimensions below the incident wavelength are displaced from their equilibrium state. As a consequence, the structure’s surface is polarized. Under the electric force of the surface charge, the dislocated electrons oscillate with a certain frequency (plasmon resonance frequency), giving rise to an enhanced electromagnetic field surrounding the nanostructures^1–3^. This field substantially augments their absorption and scattering cross-section^3^. Based on this concept, one can expect that plasmonic nanostructures enable the design of “sub-wavelength” thin layers preserving certain optical properties of the bulk material. Several scientific studies on fractal and plasmonic structures have already attempted to find ideal absorbers^3–12^ and to bring them at a “sub-wavelength” thin film scale in optodevices. In this regard, noble metal black^3–5^ represents an appreciated optical absorber material with the potential for use as a thin layer in optical sensors. Presently available optical absorbers in the wavelength region from 0.4 µm to 20 µm rely on silver black^13,14^. However, silver black displays an undesired chemical reactivity to air components^13,14^, hindering its long term stability of performance, which is important for microelectronics technology. Our alternative is to utilize platinum black, which together with a broad absorbance and low reflectance from ultraviolet to infrared region^15,16^ exhibits the required chemical stability in air. In most cases, the insertion of the optical absorber in the construction of microelectronics requires a non-aqueous media for operation and a prospective that the material can be brought into a thin-layer. Therefore, an *in situ* electro-deposition of platinum black on the microcomponents in non-aqueous media is of practical interest. The *ex situ* preparation of the platinum black layers is beneficial because it does not require conductive substrates; however, it encounters obstacles in achieving high homogeneity and highly localized deposition. In contrast to the time-consuming *ex situ* preparation, the *in situ* electrochemical method achieves a great regularity and a precise localization of the metal electro-assembled in just a few seconds. In the present study, we construct *in situ* electro-assembled layers of platinum black in isopropanol at a sub-visible-wavelength thickness and compare their properties with the micrometre scale thickness of *ex situ* platinum black trapped in a thermo-resistive resin. The obtained data show that by reducing the *in situ* constructed film to sub-visible-wavelength thicknesses, the measured reflectance in the region from the ultraviolet to the infrared remained close to that exhibited by the micrometre-sized films. This insight can be only explained by an increase in the absorption plasmonic cross-section under incident light.

## Results

### *In situ versus ex situ* platinum black layers

#### Morphology

**Figure 1:**
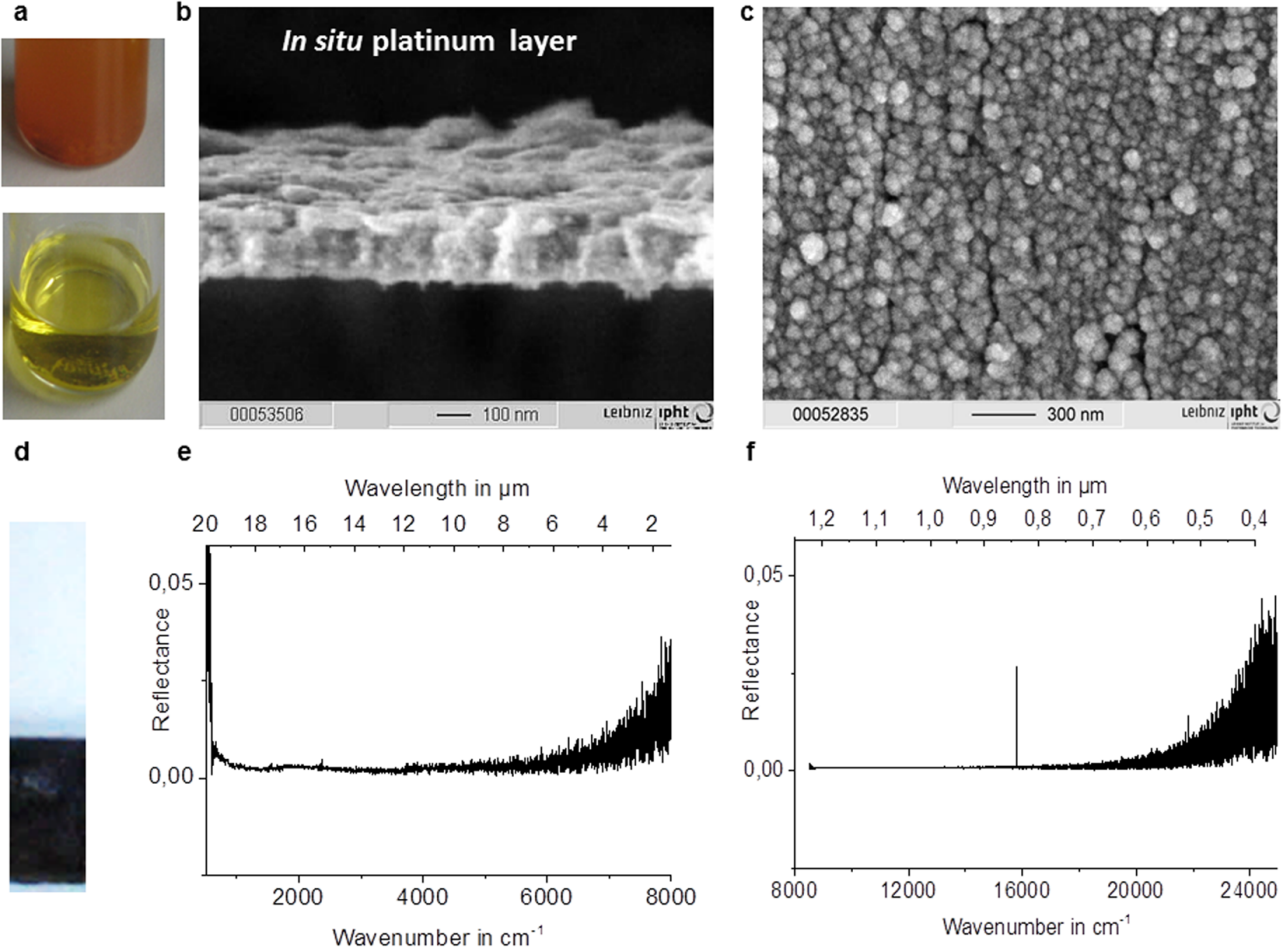
Optical properties of the *in situ* electro-assembled platinum black in nonaqueous media. (**a**) The photograph of the electrolytic bath of platinum chloride solution in isopropanol immediately after mixing (up) and after one day of dissolution (down); (**b**) Lateral view SEM image of the *in situ* electro-assembled platinum black thin layer; (**c**) Top view SEM image of the *in situ* electro-assembled platinum black thin layer; (**d**) photograph of the platinum thin layer on ITO; (**e**,**f**) FTIR reflectance spectra of the platinum thin layer in the region 400–8000 cm^−1^, 8000–25000 cm^−1^.

**Figure 2:**
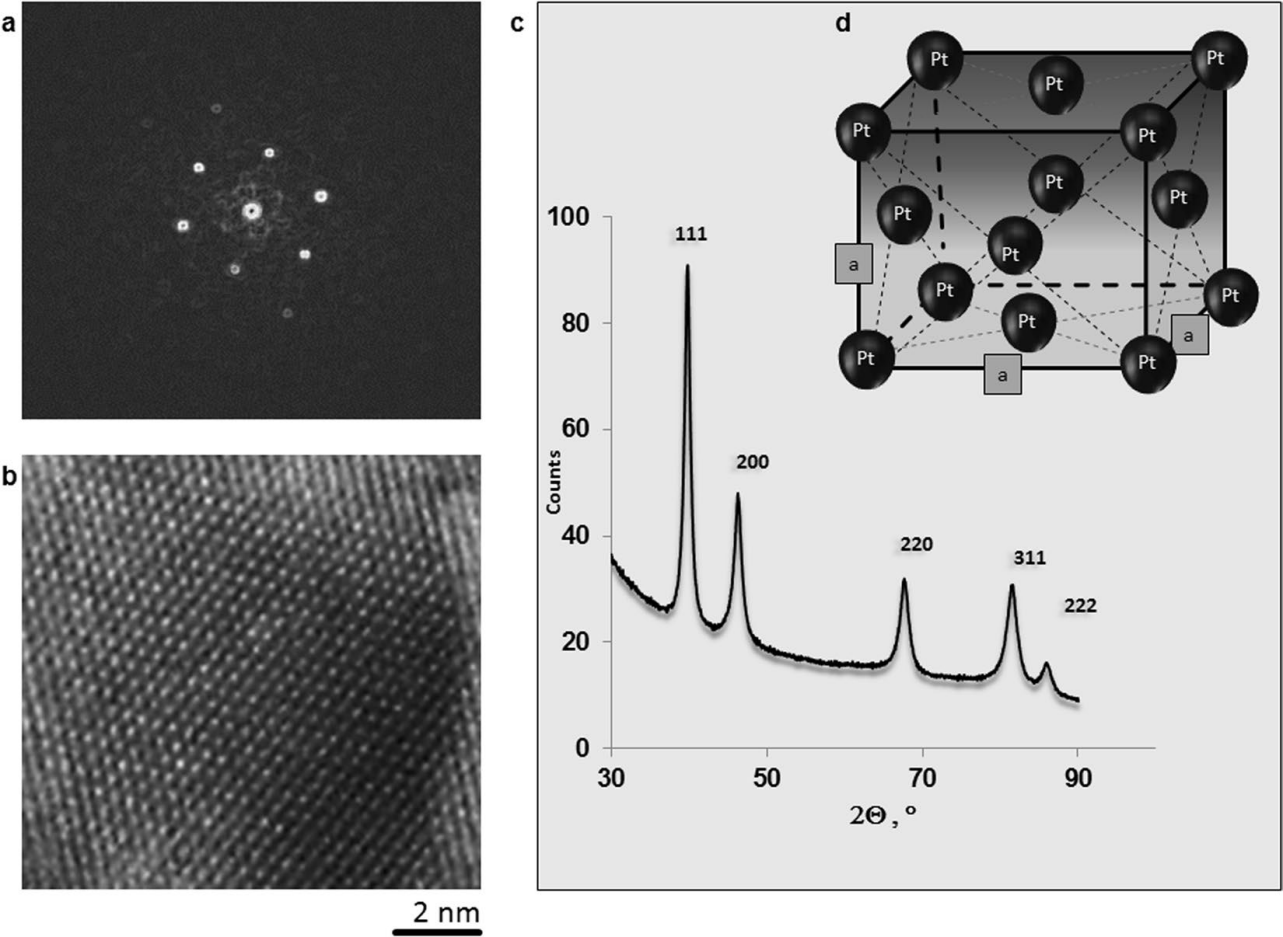
Characterization of the electro-assembled platinum black. (**a**) FFT on the area indicated in (**b**).(**b)** HRTEM of the platinum black; (**c**) XRD pattern; (**d**) The scheme of the fcc cell.

**Figure 3:**
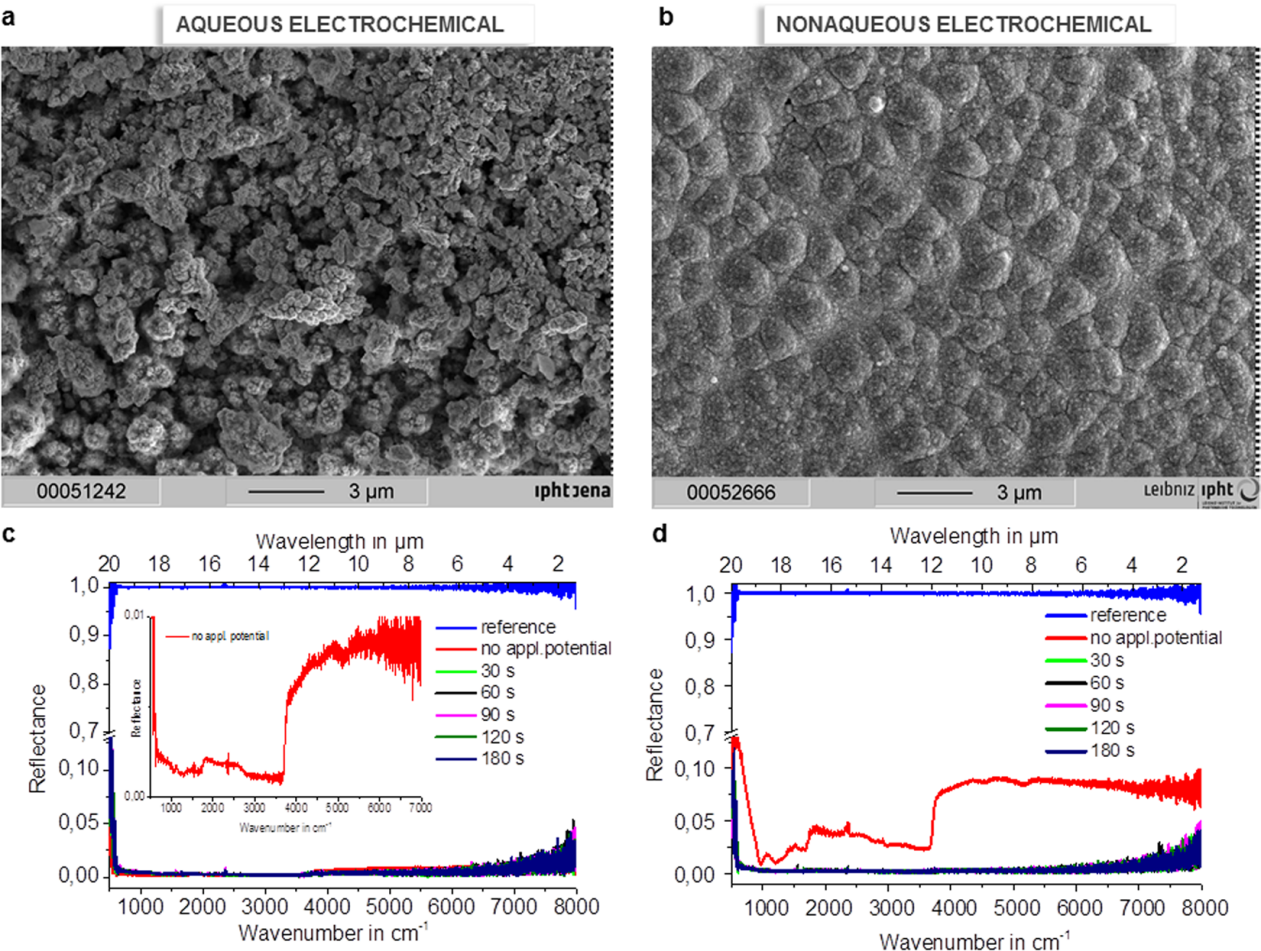
Optical assets of the *in situ* built platinum black layer. (**a**,**b**) SEM of the *in situ* electro-assembled platinum black layer on copper: (**a**) in aqueous, (**b**) in non-aqueous; (**c**,**d**) Reflectance FTIR spectra of the platinum electrodeposited on copper wires in aqueous(**c**) and non-aqueous (**d**) media at various electrolysis times. Silver mirror represents the reference for the reflectance.

**Figure 4:**
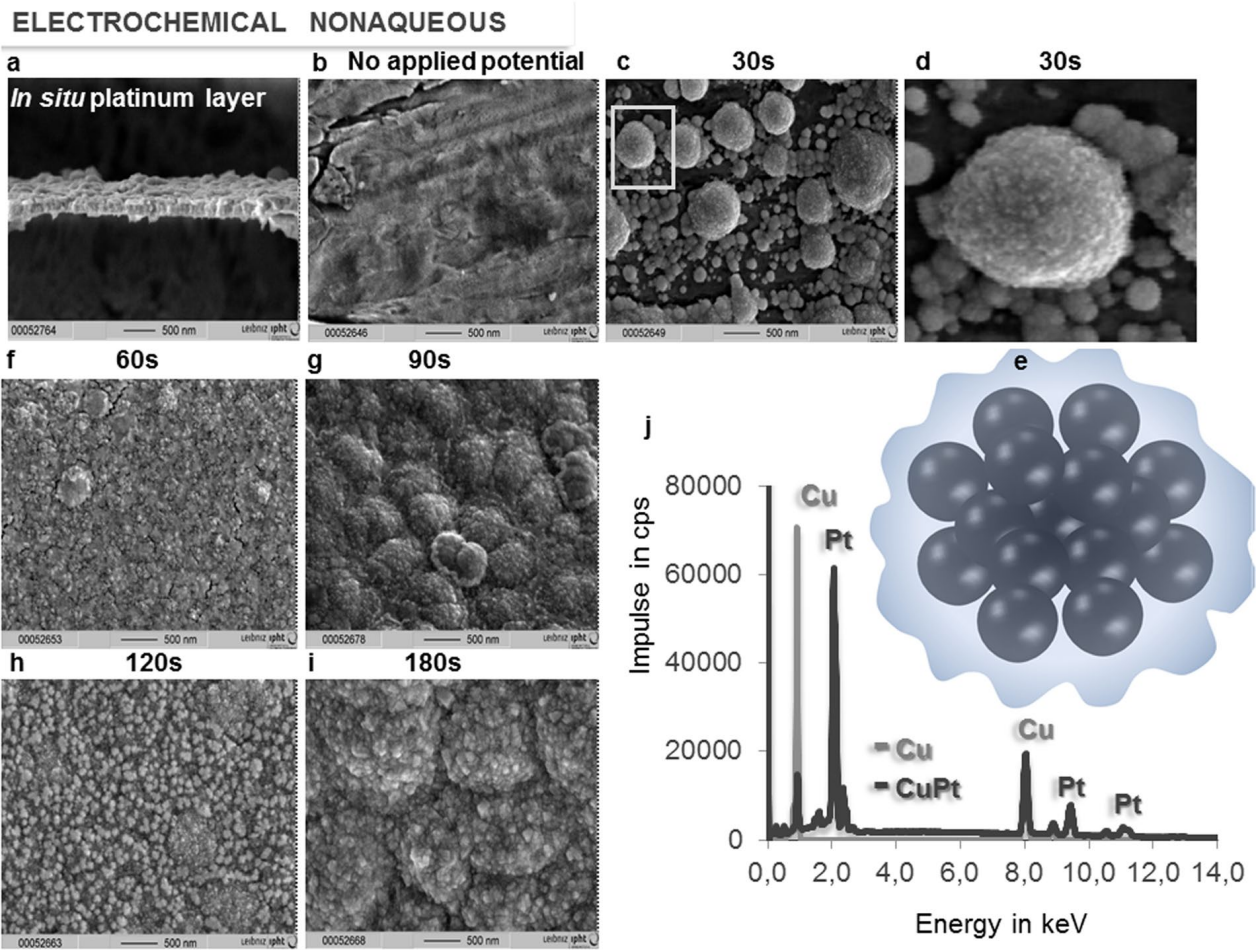
Optimizing electrodeposition time. (**a**) SEM image of the *in situ* electro-assembled platinum black layer transversal cut; (**b**–**d)**, (**f**–**h**) SEM of electrosynthesized platinum black layer on copper in non-aqueous media at various electrolysis times: 0 s no applied potential (**b**), 30 s (**c**,**d**), 60 s (**f**), 90 s (**g**), 120 s (**h**), 180 s (**i**), (**e**), the schematic representation of the structure indicated in (**d**); (**j**) EDX pattern of the copper wire (grey) and platinum layer on copper wire (black) after 300 s electrolysis time.

**Figure 5:**
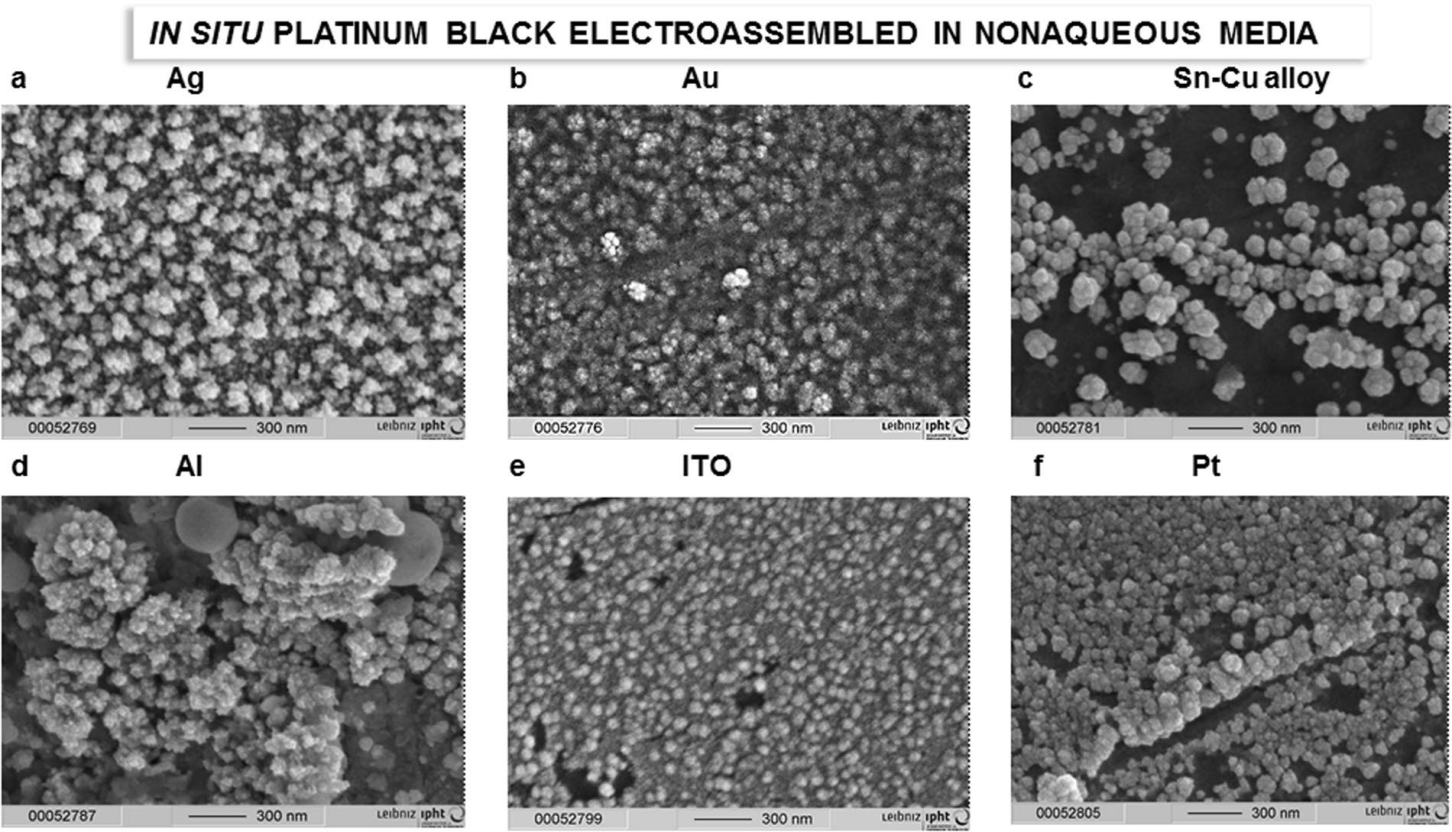
*In situ* electro-assembled platinum black thin layer in 90 s on several substrates. (**a**) silver wire; (**b**) gold wire; (**c**) Sn-Cu alloy wire; (**d**) aluminium wire; (**e)** Indium Tin Oxide (ITO) glass; (**f**) platinum wire.

The electro-synthesized platinum black powder and the *in situ* electro-assembled platinum layers in nonaqueous media were examined using scanning electron microscopy (Fig. 1), high resolution transmission electron microscopy with Fast Fourier Transform image analysis (Fig. 2a,b), and X-ray diffraction (XRD) (Fig. 2c,d). Electron micrographs show a uniform layer (Fig. 1b), with the porosity strongly dependent on the cathodic materials and other electrolysis conditions (Figs 3–5). The 100-nm thick layers similar to the one illustrated in Fig. 1b, are grown on wires of copper, platinum, aluminium, silver, Sn-Cu alloy, gold and on indium tin oxide (ITO) glass plate in a non-aqueous solution of PtCl_4_ at a current density of 0.1 A/cm^2^. The layers consist of platinum crystals with a polycrystalline face centred cubic structure (Fig. 2d), with a small texture leading to an increase in the 111 reflection (Fig. 2c). Using the Scherrer formula and evaluating the spectrum shown in Fig. 2c, the crystallite size is estimated to be approximately 10 nm. This result is an average value over all spatial directions. Owing to the high background in the spectra, the contribution of the smallest dimension (i.e., largest FWHM) may show a higher measuring error. The crystalline nature of platinum was confirmed by HRTEM and fast Fourier transform image analysis (Fig. 2a,b). In the EDX spectra recorded at E_0_ = 20 keV, the peaks related to platinum are clearly identified (Fig. 4j).

#### Light reflectance/transmittance characteristics

Upon illumination of a medium, the radiant flux can pass through this medium (transmission), can be absorbed by the medium or can be returned (reflection) inside of the medium (volume reflection) and at the medium boundary (surface reflection). Based on the conservation of energy, the sum of reflectance, transmittance and absorbance equals unity. However, both reflection and transmission can undergo scattering (diffusion), which refers to the deviation of the radiant unidirectional beam into several other directions. The resultant radiation beam consists of specular (regular) components and diffuse components of the reflection and transmission. The diffuse components can cause that the measured spectra depend on the geometry of the collection optics of the detector system^17^. The radiation beam follows geometrical laws of optics only in the ideal case when no diffusion arises and results in a pure regular reflection and transmission. The dispersive component of the radiant flux can cause a false decrease of the regular reflection intensity; however it is mostly under the detector detection limit. The samples were illuminated with a tungsten lamp, and the spectra were collected using a standard NIR RT-Si Diode detector, operating at room temperature, and a FTIR detector with liquid nitrogen cooled mercury cadmium telluride (MCT) diode. The measurements were performed at the source aperture, collection mirror velocity and angle that show minimal noise. The spectra are biased by the instrument radiometric accuracy. The absorbance, calculated by subtracting from 100% the transmittance and the reflectance, contains the error given by the detection limit of the detectors (§Methods) and those caused by neglecting the dispersive component of the radiant flux.

**Figure 6:**
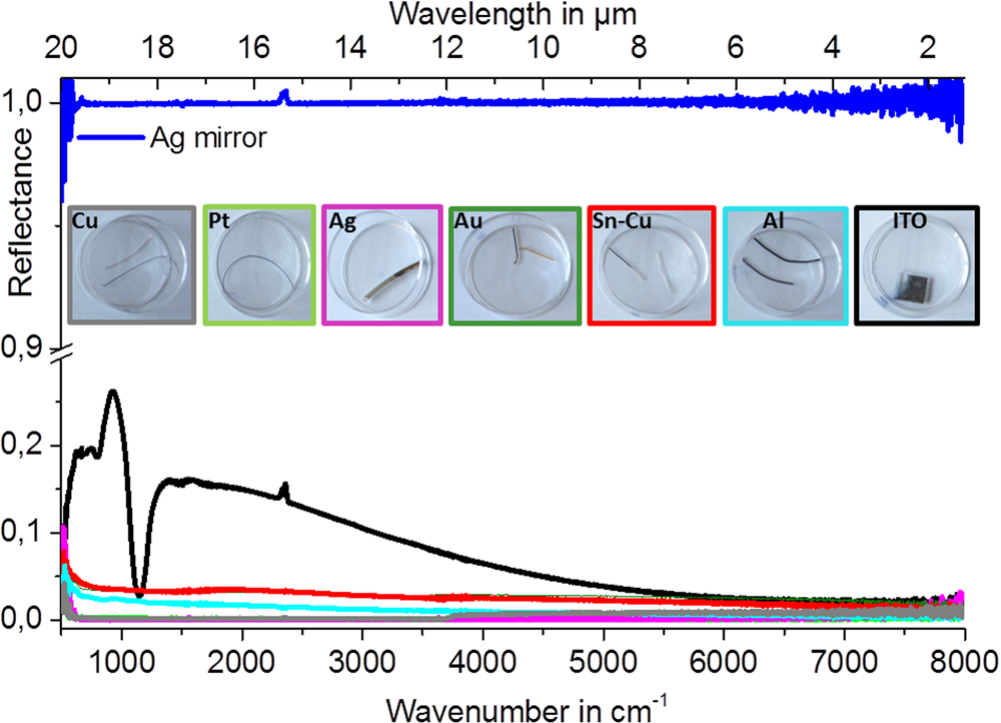
Optical properties of the i*n situ* electro-assembled platinum black thin layer on different substrates. The FTIR reflectance spectra referred to silver mirror of the platinum black layers achieved in 90 s in non-aqueous media on ITO (black line), tin-copper alloy (red line), aluminium (blue cyan), copper (grey), gold (olive), platinum (green), silver (magenta) (the other electrolysis conditions described in §Methods); the insets illustrate the photographs of the platinum black on different substrates indicated on the panels.

**Figure 7:**
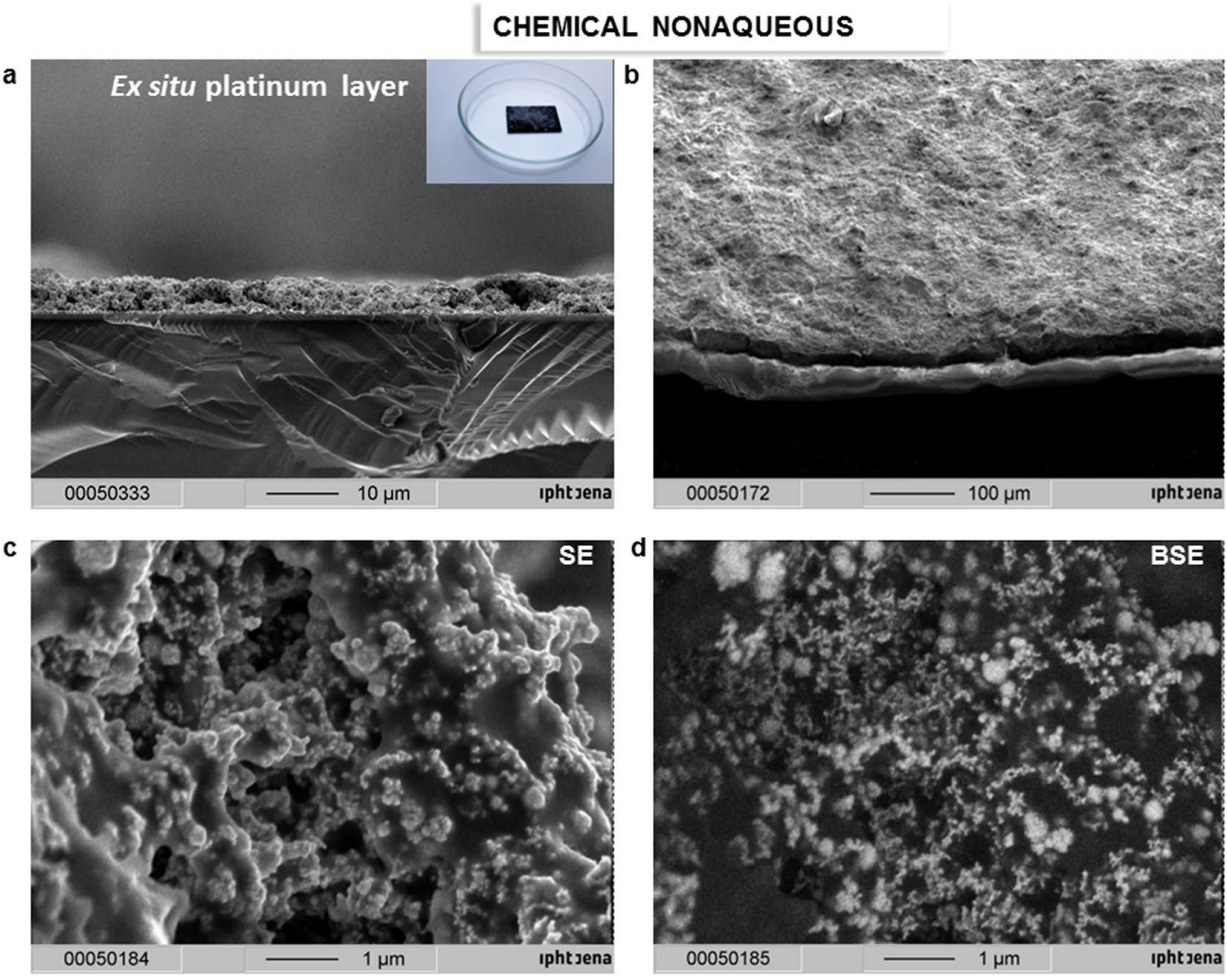
*Ex situ* platinum black layer. (**a**) SEM image of the transversal cut platinum black layer *ex situ* built up; the inset photograph shows the silicon wafers covered with lack entrapped platinum black (**b**) Top-view SEM image of the platinum black; (**c**–**d**) SE-BSE images of the platinum black layer on identical area; resin-black area and platinum-white area in BSE image.

**Figure 8:**
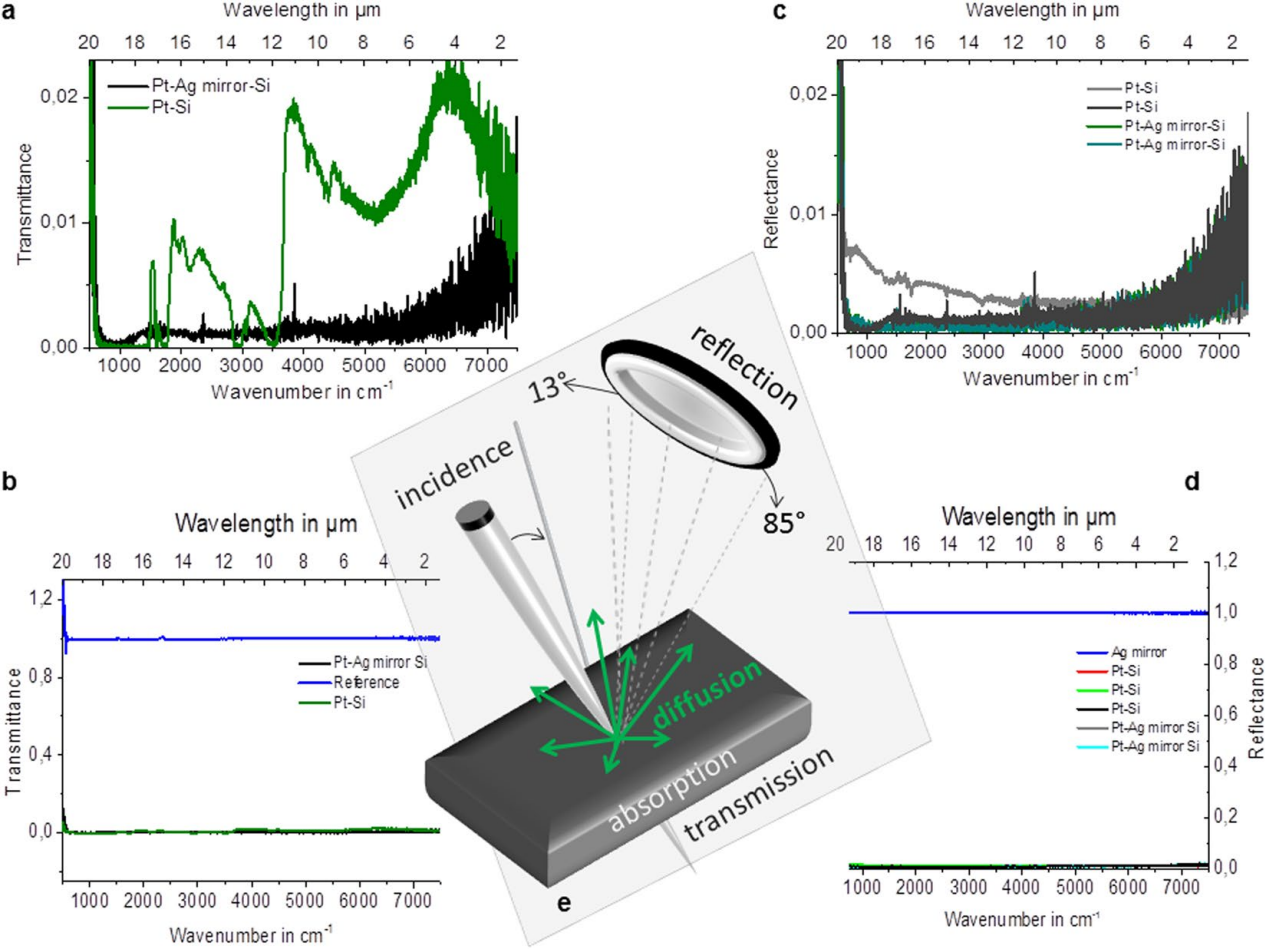
FTIR of *ex situ* platinum black. (**a**–**d**) Transmittance (**a**,**b**) and reflectance (**c**,**d**) FTIR spectra of the platinum deposited on Si substrate and on silver mirror-Si substrate, respectively. Silver mirror represents the reference for the reflectance and the air, for transmission, respectively. (**e**) The schematic representation of the radiant flux distribution upon illumination of a medium; the setup permits to adjust the collection mirror angle in the reflection unit in the interval 85°–13° while the incident angle remains unchanged.

The *in situ* (Figs 1b,c, 4, 5, 6) and *ex situ* (Fig. 7) platinum black layers are examined by the mean of FTIR spectroscopy. Reflectance spectra in the infrared region of the *in situ* (Figs 1e,f, 3c,d, 6) and *ex situ* platinum black layers are separately investigated (Fig. 8a–d). Every spectrum is an average of 28 spectra. The reflectance spectra recorded from 24000 to 8000 cm^−1^ (corresponding to 0.4 to 1.25 µm wavelength) and from 8000 to 500 cm^−1^ (corresponding to 1.25 to 20 µm wavelength) presented in Figs 1and 3 show constantly lower values than 0.05 referred to silver mirror. The layers at various electrolysis times from 30 s to 180 s were also examined by FTIR (Fig. 3c,d). Even for the electrolysis time of 30 s, the absorbing layer exhibits a mean reflectance below 10% from 1.25 µm to 20 µm. Electrolysis times longer than 300 s result in layer disruption, with the reflectance still maintained at a low level. The *ex situ* prepared chips, which consist of silicon and silver mirror-covered platinum black entrapped in a thermoresistive resin with an overall thickness of 5 μm (Fig. 7), exhibit low reflectance and transmittance in the entire region of investigation (Fig. 8). These data show that the *in situ* deposition of platinum black reaches layers with optical properties similar to the *ex situ* micrometre thickness layers, being promising for the application in the fabrication of optoelectronics.

### Layers homogeneity and roughness

SEM analysis of the platinum black embedded in thermo-resistive resin and immobilized on silicon on silver mirror substrates (§Methods) shows a micrometre-sized layer of platinum (Fig. 7a) and a porous morphology with a roughness of approximate 200 nm. SEM indicates a relatively homogeneous distribution of platinum crystals into the resin (Fig. 7d). Instead, the *in situ* electro-assembled platinum layers exhibit superior homogeneity with a roughness lower than 50 nm at the electrolysis times between 60 and 180 s (Figs 3–5). At electrolysis times longer than 180 s, the layers thickness reaches approximately 500 nm. Below 60 s, the layers are not completed at the specified concentration and current density. The layer growth can be accelerated by increasing the concentration and current density with the drawback of the layer outpouring. The optimum layer homogeneity and optical properties were obtained at an electrolysis time of 90 s at an electrical current density of 0.1 A/cm^2^. The method is influenced by the electrolyte composition, electrolysis time, current density and substrate composition and structure (Fig. 5). Similar working parameters lead to different results as a function of the substrate. Higher homogeneity of the *in situ* formed platinum black layer is obtained on silver, gold, and copper (Figs 4, 5a,b), and medium homogeneity is obtained on platinum and ITO (Fig. 5e,f), while on aluminium and tin-copper alloy (Fig. 5c,d), platinum black growth requires a longer time (180 s) to achieve similar homogeneity. The FTIR spectra in the region 500–8000 cm^−1^ of the platinum layers on different substrates are illustrated in the diagram of the Fig. 6. The platinum black layers on aluminium and tin-copper alloy exhibit a reflectance of approximate 0.05 compared to the silver mirror (reflectance = 1). The platinum black deposited on ITO shows reflectance smaller than 0.05 in the region 5000–8000 cm^−1^, reflectance of 0.05 ÷ 0.2 from 500 cm^−1^ to 5000 cm^−1^ except the peak of 0.32 reflectance at 1000 cm^−1^. The platinum black -on platinum, gold, silver, copper presents a lower reflectance than 0.05 in the whole investigated region.

## Discussions

### Electrochemical formation of platinum black thin layer in non-aqueous media

It was demonstrated that platinum black can be electroc–hemically synthesized in non-aqueous media^16^. On this basis, a sub-visible-wavelength thick layer of platinum black in isopropanol is electrochemically constructed (§Methods). The anodic material (platinum wire) ensures stability against oxidation while isopropanol is oxidized to acetone as follows:

1 $$2{({{\rm{CH}}}_{{\rm{3}}})}_{2}{\rm{C}} \mbox{-} {\rm{OH}}\to 2{({{\rm{CH}}}_{3})}_{2}{\rm{C}}={\rm{O}}+2{{\rm{H}}}^{+}+2{{\rm{e}}}^{-}$$

At the cathode, the reduction of platinum ions occurs:

2 $${{\rm{Pt}}}^{4+}+4{{\rm{e}}}^{-}\to {\rm{Pt}}\downarrow \,{\rm{along}}\,{\rm{with}}\,{\rm{gaseous}}\,{\rm{hydrogen}}\,{\rm{formation}}:2{{\rm{H}}}^{+}+2{{\rm{e}}}^{-}\to {{\rm{H}}}_{2}\uparrow $$

The proposed cathodic reaction sequences are described in equation (3):

3 $${{\rm{Pt}}}^{4+}+2{{\rm{e}}}^{-}\to {{\rm{Pt}}}^{2+}\,{\rm{and}}\,{{\rm{Pt}}}^{2+}+{{\rm{H}}}_{2}\to {\rm{Pt}}\downarrow +2{{\rm{H}}}^{+}$$

**Figure 9:**
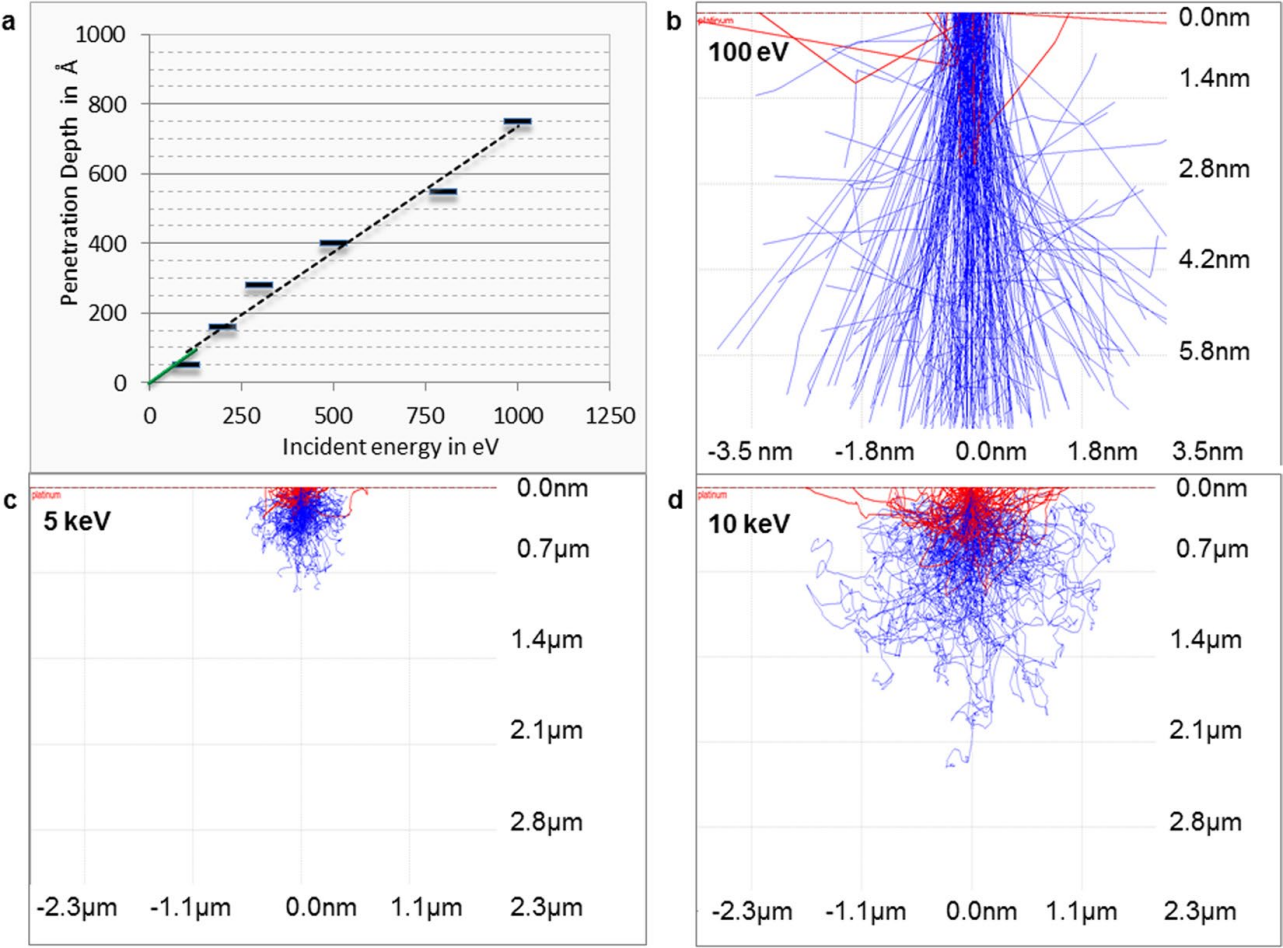
Monte-Carlo simulation of electron trajectories in platinum black of ρ = 1.6 g/cm³. (**a**) Simulated penetration depth (5% in platinum at various electron energy, the green marked line represents the plot extrapolation in the domain 0.06199 eV corresponding to the 20 µm and 0.99187 eV corresponding to the 1,25 µm); (**b**–**d**) simulated electron trajectories at 100 eV(**b**), 5 keV (**c**) and 10 keV (**d**).

Before applying an electrical potential difference between the electrodes, by immersing the metal (i.e. copper wire) into an electrolyte containing the salt of a more noble metal (i.e. PtCl_4_) a sudden deposition of the more noble metal occurs. This is the reason that at 0 s electrolysis time the reflectance is already low, however the layer is not observed as black. The SEM image of the copper at 0 s electrolysis time when no electrical potential difference is applied (Fig. 4b) shows no evidence of porous platinum; nevertheless an ultrathin layer which decreases the metallic high reflectance is present. Later on, when a current flows through the electrochemical cell, the applied potential, partially used by the redox process, will differently drop across the solution in aqueous versus non-aqueous media (with an iR value), and along the polarization process to attain the potential at which the faradaic processes take place (with an over potential η value)^18^. The potential drop depends on the conductive properties of the electrolyte, as well as, of the cathodic material. The electrical conductivity of the nonaqueous electrolytic bath being smaller than the one for the aqueous bath, an increase of the potential drop across the nonaqueous media occurs. In addition, the non-faradaic processes such as adsorption and desorption of the electroactive species on the electrode, and the ion mobility will differ from aqueous to non-aqueous media, with consequences on the electrodeposition process of platinum black. The benefit of slower electrochemical processes in nonaqueous media is that the formed platinum black layers are better defined, thinner and more homogeneous (Fig. 3b versus Fig. 3a). When we look at the SEM images in the Fig. 3a,b we observe differences in homogeneity and in the shape of the structures. Instead, when we spectroscopically examine the black formed layers both of them show low transmittance and low reflectance from UV to the IR region (Fig. 3c,d). Analysis of the *in situ* formed films recorded at progressively increased electrolysis times shows that the deposition starts at certain points on the surface, most likely on its defects, where a “pile”-like structure with a height of approximately 20 nm is created (Fig. 4c), which then is extended along the current flow lines towards covering the whole surface. After completing one layer, the next one starts to grow (Suppl. Information) by a similar mechanism. This is the case of nanolayer by nanolayer growth as estimated from SEM images in the cut of the films. The dimension of the layers depends on cathodic materials, electrolyte composition, and applied potential. In non-aqueous media the layers are thinner than in aqueous media. The optimum uniform platinum crystalline layers of 100 nm thickness are formed in 90 s. Although the crystal unit lattice is the same as the database value of metallic platinum, the density value of the porous assembly is approximately one order of magnitude lower at ρ = 1.6 g/cm^3^ than the value for the metallic platinum density. This detail suggests that light can find diffractive pathways to arrive inside the nanostructure and to stimulate and dislocate the electrons from their states, creating high local near-fields that affect the absorption cross-section. This can explain the light reflectance characteristics exhibited by the *in situ* assembled platinum layers of sub-incident wavelength thickness (Figs 3c,d, 6). The simulated electron trajectories and the simulated penetration depth (5%) of the various electron energies in porous platinum show a linear decrease of the penetration depth with the energy (Fig. 9). By extrapolation, the light of energies from 0.99187 eV (corresponding to the 1.25 µm) to 0.06199 eV (corresponding to the 20 µm) will allow a penetration depth of a few angstroms. This result suggests that low energy light enables dislocation of the superficial crystals conduction electrons and generates their oscillation into a scattering volume of a certain cross-section. The calculated cross-section values (Suppl. Information) (*σ*_*scatt*_) under the incidence of 400 cm^−1^ and 25000 cm^−1^, respectively, vary from 0.836 10^−7^ pm^2^ to 1.27 pm^2^, for sphere of 10 nm and from 5.35 pm^2^ to 81.64 10^6^ pm^2^ for spheres of 200 nm. The data shows differences in cross-section of seven orders of magnitude when the incident light switches from 400 cm^−1^ to 25000 cm^−1^ and suggests that the dislocated electrons generate vibrating clouds of different dimensions around the particles. The particles associate into a porous structure (Fig. 4e); when in these structures the crystals are closer than 0.5 nm to each other the quantum-mechanical coupling mechanisms act^19^. Therefore, these vibrating clouds also interfere forming a global vibrating electrical “atmosphere” of the nanostructures (Fig. 4e). To mathematically describe more rigorously the increased scattered field cross-section, the Suppl. eq. 1 needs to be completed with terms including the density of the target area, the target atomic number, the incident angle, the kinetic energy and the irregularity of the structure^20^. In the absence of a rigorous mathematical support, based on the experimental data, one assumes that the global scattering volumes of all involved wavelengths coupled with the inertia effect can explain the optical properties of the electro-assembled sub-wavelength layers: a broad low reflectance and transmittance of the electromagnetic waves. The *in situ* achieved electrochemical preparation of the platinum black thin layers is desired in the fabrication of optoelectronics. We proved the electrosynthesis of platinum black on different cathodic materials in aqueous and non-aqueous solution of PtCl_4_. Although the obtained platinum films differ morphologically as a function of cathodic material and electrochemical bath composition, they all show low transmittance and low reflectance of the infrared radiation even at the sub incident wavelength thickness. The above mentioned optical properties combined with a renowned physicochemical stability make platinum black an outstanding candidate for the construction of stable optoelectronics.

## Methods

### *In situ* electro-assembling of platinum black thin layer on various substrates

A 20 mL electrochemical cell, equipped with two electrodes connected to a continuous current source, which assures the electrical current density of 0.1 A/cm^2^, was used for electrolysis. To synthesize platinum black in isopropanol media,  0.05 g PtCl_4_ and 0.008 g Pb(CH_3_COO)_2_ dissolved in 10 mL isopropanol was used as electrolytic bath (Fig. 1a-up).The dissolution takes several hours at room temperature. During this process, the colour turns from brownish to yellow (Fig. 1a-down); therefore, the solution is prepared with one day before the electrolysis. Pt wire serves as the anode, and the cathode has been successively selected as platinum wire, aluminium wire, silver wire, Sn-Cu alloy wire, gold wire and plate, indium tin oxide (ITO) glass, or copper wires. Electrolysis times varying from 30 s to 300 s have been examined, and it was found that 90 s represents the optimum one for compact layers of 100 nm thickness. In aqueous media the electro-assembling of platinum black on cathodes of different materials is prepared as described in our previous work^16^. Electrolytic bath: 0.05 g PtCl_4_ and 0.008 g Pb(CH_3_COO)_2_ dissolved in 10 mL water.

### Non-aqueous preparation of platinum black

0.1 g platinum salt (Platinum IV chloride: PtCl_4_ 5H_2_O, MW 336.90 g·mol^−1^Merck KGaA S5505147/003/8.07347.0005) was dissolved in 10 mL isopropanol, and separately, 0.2 g of reduction agent (Sodium tetrahydroborate NaBH_4_, MW 37.83 g·mol^−1^Roth, Art. No. 4051.1) was dissolved in 2 mL isopropanol at 18–20 °C. NaBH_4_ solution is added in drops to the platinum salt solution. A black colour of the dispersion indicates the end of the synthesis^16^.

### *Ex situ* assembly of platinum black layer

500 mg of as-prepared platinum black precipitate is mixed with 100 µL of AZ 1514 type lack and then placed on Si and Si-Ag mirror wafers. The samples were investigated by Fourier-transform-infrared spectroscopy (FTIR) and scanning electron microscopy (SEM).

### Scanning Electron Microscopy (SEM)

SEM measurements were performed with a field emission microscope JSM-6300F (JEOL, Tokyo, Japan). The energy of the exciting electrons was mostly 5 keV. Beside the detector for secondary electrons (SEI), Everhart-Thornley type, the system is equipped with different detector types (semiconductor and YAG type) for backscattered electrons.

### Transmission Electron Microscopy (TEM)

Dried samples deposited on a carbon coated 400 mesh copper grid were examined by the mean of High Resolution Transmission Electron Microscopy (HRTEM) using a TEM JEOL JEM-3010 operating at 300KeV.

### X-Ray diffraction

The X-ray diffraction analysis has been performed with an X’pert Pro Instrument (PANanalytical, Almelo, Netherlands) using Cu-Kα1, 2 radiation. The Scherrer equation was used for the determination of the crystallite sizes.

### Infrared Spectroscopy

The visible, near infrared and infrared spectra of the platinum layers were measured with the Reflectance Mode and the Transmission Mode in a FTIR-Spectrometer (Bruker Instrument), respectively. The spectra were recorded with a resolution of 1 cm^−1^ in the spectral range 8000–500 cm^−1^. The samples were illuminated with a tungsten lamp and the spectra were collected with a standard NIR detector: RT-Si Diode, which operates at room temperature (detection limit NEP: <10^–14^WHz^−1/2^) and a FTIR detector with Mercury Cadmium Telluride (MCT) diode (D*: >2 × 10^10^cmHz^1/2^W^−1^) liquid nitrogen cooled. The Bruker 80 v setup permits to adjust the collection mirror angle in the reflection unit in the interval 85°–13° while the incident angle remains unchanged (Fig. 8e). The measurements were performed at the source aperture, collection mirror velocity and angle that show minimal noise.

## Electronic supplementary material

Supplementary Information
