# Supplementary material for: Optical Assets of In situ Electro-assembled Platinum Black Nanolayers
Source: Sci Rep. 2017 Nov 2;7:14955. doi: 10.1038/s41598-017-14630-3 (PMC5668411; doi:10.1038/s41598-017-14630-3)

Supplementary Information

Optical Assets of *In situ* Electro-assembled Platinum Black Nanolayers

S.E. Stanca[1*], F. Hänschke[1], G. Zieger[1], J. Dellith[1], A. Ihring[1], A.Undisz[2], H.-G. Meyer[1,*]

Scattering cross-section

The scattering cross-section for spherical nanoparticles was roughly calculated using the classical equation 1:

$$\sigma_{scatt} = \frac{8\pi}{3} R^6 k^4 \left(\frac{\varepsilon_{particle} - \varepsilon_{medium}}{\varepsilon_{particle} + 2\,\varepsilon_{medium}}\right)^2 \qquad (1)$$

where ε_{medium} is the permittivity of the medium (=1 for air); $\varepsilon_{particle}$ is the relative permittivity of the particle:

$$\varepsilon_{particle} = 1 - \frac{{\omega_{plasma}}^2}{\omega^2} \qquad (2)$$

ω_{plasma} is the plasma frequency; ω is the frequency of the electromagnetic incident radiation, R-radius of the particle in nm, k is the wavenumber in cm^{-1}. Under the assumption that the permittivity tends to zero for nanoparticles, the eq. 1 becomes:

$$\sigma_{scatt} = \frac{8\pi}{3} R^6 k^4 \left(\frac{1}{2}\right)^2 = \frac{8\pi}{12} R^6 k^4 = \frac{2\pi}{3} R^6 k^4 \qquad (3)$$

For the crystallite size, with the diameter of 10 nm (data from XRD analysis), R=5 nm, R^6=15.6 10^3 nm^6 we obtained the following cross-section values:

1) for an incident light of 400 cm^{-1}, k=400 cm^{-1}, k^4=25.6 10^9 $(cm^{-1})^4$

σ_{scatt} =0.836 10^{-7} pm^2

2) for an incident light of 25000 cm^{-1}, k=25000 cm^{-1}, k^4=3.9 10^{17} $(cm^{-1})^4$

σ_{scatt} =1.27 pm^2

The crystallites form larger structures from 50 to 400 nm as indicated in the SEM images (Fig. 4d-e).

For a spherical structure of 200 nm diameter, R=100 nm, R^6=10^{12} nm^6 we obtained the following cross-section values:

1) for an incident light of 400 cm^{-1}, k=400 cm^{-1}, k^4=25.6 10^9 $(cm^{-1})^4$

σ_{scatt} =5.35 pm^2

2) for an incident light of 25000 cm^{-1}, k=25000 cm^{-1}, k^4=3.9 10^{17} $(cm^{-1})^4$

σ_{scatt} =81.64 10^6 pm^2

Steps in electro-assembling of porous platinum

Completion of the first layer of porous platinum on copper:

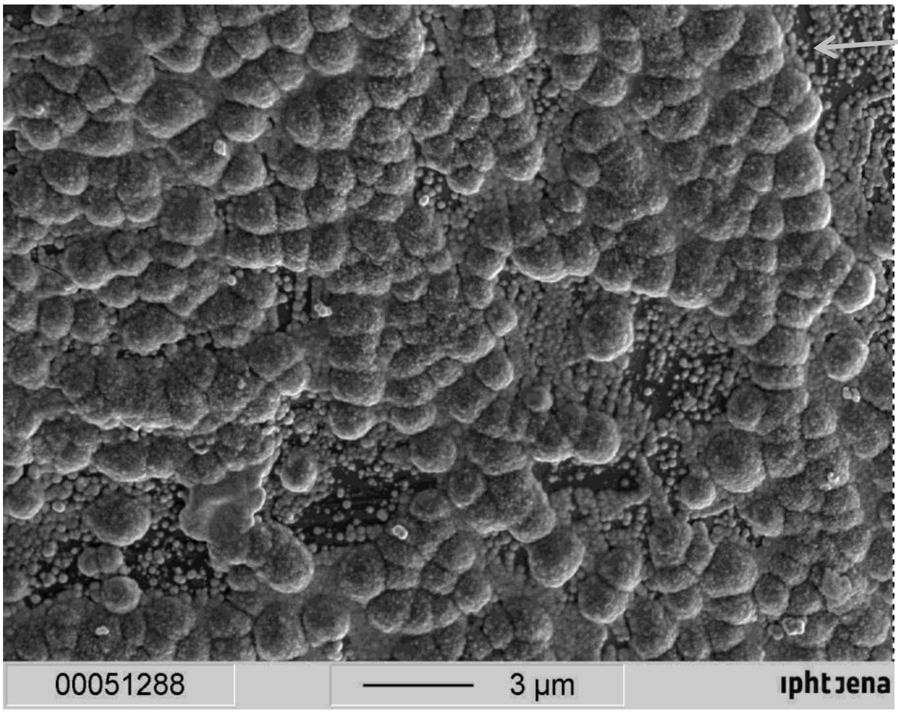

Initiation of the layer via “pile” like structures

The initiation of the second layer on an existing platinum layer on copper:

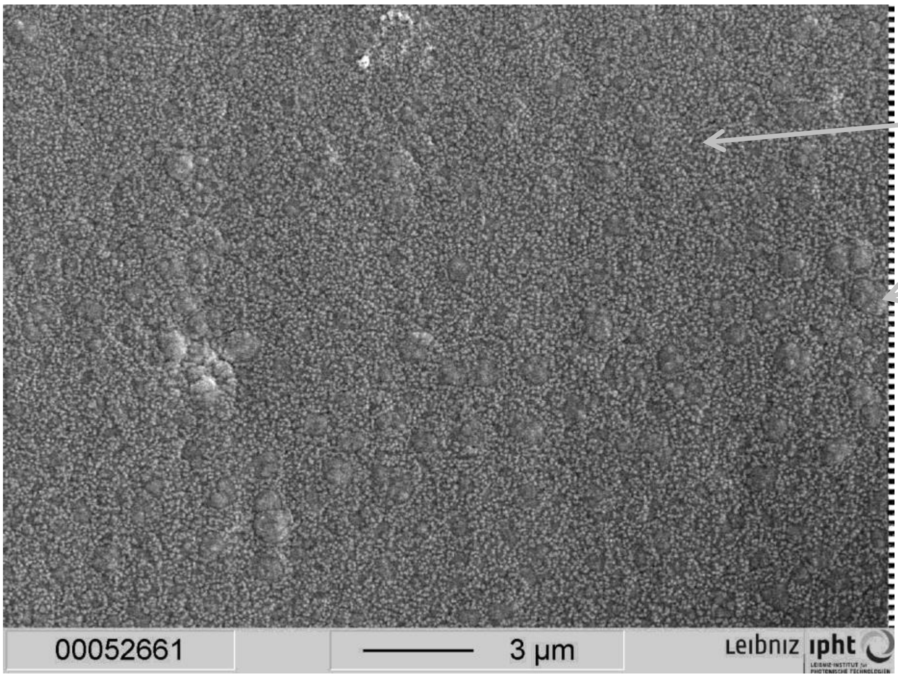

Initiation of the second layer via “pile” like structures

Initial platinum layer

The second platinum layer completed:

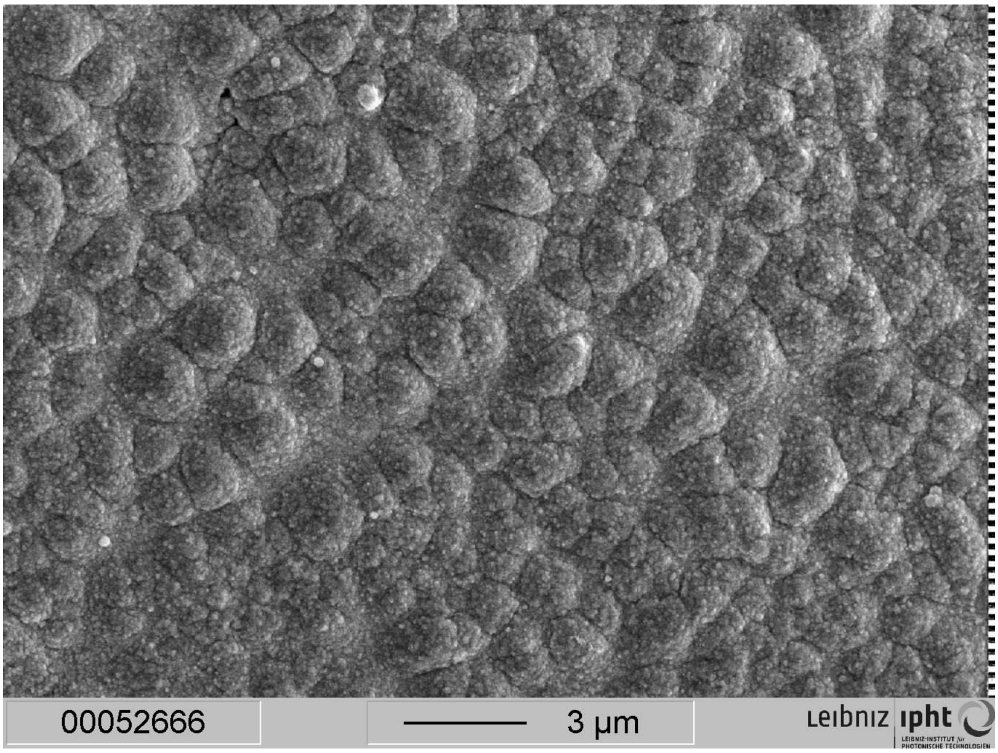

Supplement: Supplementary file 1 — Supplementary Information [file 41598_2017_14630_MOESM1_ESM.pdf]
